# Screen-Printed Voltammetric Sensors—Tools for Environmental Water Monitoring of Painkillers

**DOI:** 10.3390/s22072437

**Published:** 2022-03-22

**Authors:** Katarzyna Tyszczuk-Rotko, Jędrzej Kozak, Bożena Czech

**Affiliations:** Faculty of Chemistry, Institute of Chemical Sciences, Maria Curie-Skłodowska University in Lublin, 20-031 Lublin, Poland; jedrekkozak@onet.pl (J.K.); bczech@hektor.umcs.lublin.pl (B.C.)

**Keywords:** screen-printed sensor, voltammetric analysis, painkillers, environmental water monitoring

## Abstract

The dynamic production and usage of pharmaceuticals, mainly painkillers, indicates the growing problem of environmental contamination. Therefore, the monitoring of pharmaceutical concentrations in environmental samples, mostly aquatic, is necessary. This article focuses on applying screen-printed voltammetric sensors for the voltammetric determination of painkillers residues, including non-steroidal anti-inflammatory drugs, paracetamol, and tramadol in environmental water samples. The main advantages of these electrodes are simplicity, reliability, portability, small instrumental setups comprising the three electrodes, and modest cost. Moreover, the electroconductivity, catalytic activity, and surface area can be easily improved by modifying the electrode surface with carbon nanomaterials, polymer films, or electrochemical activation.

## 1. Introduction

Increased production and consumption of over-the-counter drugs such as painkillers, e.g., diclofenac, ibuprofen, naproxen, ketoprofen, or acetaminophen, are connected with their increased excretion and presence in wastewater from homes and hospitals [1,2,3,4,5,6,7,8]. There are different sources for the contamination of surface and underground waters with drug residue, including the pharmaceutical industry, farming and veterinary, healthcare centers, and households (incorrect waste management in case of expired drugs). Some residues of the pharmaceutical substances, together with sewage, enter wastewater treatment plans (WWTP), which unfortunately are not adjusted to degrade those highly specific compounds [9,10]. Interestingly, about 2000 active pharmaceutical ingredients are administered worldwide [11]. The greatest input of pharmaceuticals into the environment have, however, treated wastewater [12,13,14,15]. It was established that 30–90% of oral doses are excreted as active substances (Table 1) [16]. Conventional WWTP are not designed to remove pharmaceuticals; therefore, the removal rates vary (Table 1). The fate of pharmaceuticals in WWTP and the environment is affected by the pharmaceutical properties, including their persistence and connected half-life. Solubility, transformation products, bioaccumulation potential, and, first of all mobility, affect their toxicity [16].

Insufficient removal from wastewater influents was a main background for the application of various wastewater treatment methods: filtration [17], adsorption [18,19,20,21], Advanced Oxidation Processes [22,23,24,25], UV [26,27], ozonation [28,29,30,31], H_2_O_2_ [32,33], photocatalysis [34,35,36,37,38,39,40], Fenton and photo-Fenton process [41,42,43], electro-catalysis [44,45], electro-Fenton [46] etc.

Although the reported concentrations of pharmaceuticals in the environmental matrices are generally low—usually less than 1 μg L^−1^, but their huge usage and abundance in the environment make the authorities worried about the long-term impact on animals and humans (Table 1) [47]. Although there are no requirements to detect and limit the concentration of pharmaceuticals in wastewater and water, some of them have been identified as a priority for further study, including paracetamol and diclofenac [48].

Despite the large differences in the removal rates, it is known that sorption, adsorption, sedimentation, and biotransformation in WWTP occurred [49,50,51,52,53,54]. Hydrophobic or electrostatic interactions are responsible for the sorption of drugs [55]. However, highly hydrophilic acidic drugs such as acetylsalicylic acid, ibuprofen, ketoprofen, naproxen, and diclofenac (pKa 4.2–4.9) are not sorbed and remain in the water [50]. The other negative effect can arise from the presence of metabolized (mainly its hydroxy and carboxy derivatives) drugs as they can form conjugates with similar or even increased toxicity in comparison to the parent drug [6]. It was established that the presence of drugs in the water revealed significant oxidative stress and caused histological changes in *Cyprinus Carpio* tissues [56] or disrupted microalgal growth.

The presence of those substances in surface waters has a toxic action on fish and other water organisms and can cause an increase in the incidence of some diseases, e.g., cancer (female sex hormones). The presence of antibiotics in water is connected with the observed increase in drug resistance of various microorganisms, even pathogenic ones. Constant exposure to pharmaceuticals in drinking water has endangered the most sensitive groups such as infants, the elderly, or patients with kidney, liver failure, or cancer. Due to the presence of estrogens in the water, feminization of male individuals and an increase in the incidence of breast and testicular cancer are noted [57,58,59]. Some of the pharmaceuticals present in water (i.e., anticancer drugs) can penetrate the blood-placenta barrier revealing teratogenic and embryotoxic effects endangering pregnant women in particular [60,61].

Induction of oxidative stress is connected with the production of reactive oxygen species (ROS) (such as hydroxyl radicals (^•^OH), superoxide radicals (O_2_^•−^), and hydrogen peroxide (H_2_O_2_) responsible for peroxidation of membranes’ polyunsaturated fatty acids and proteins [62]. In the studies considering the 98 pharmaceuticals detected in different water matrices (treated wastewater, surface water, and groundwater), it was established that 11 out of 49 pharmaceuticals were found to exert human health risk from ingesting contaminated surface water of India [63]. The growing problem of contamination of the water environment forces the use of various methods of removing pollutants and constant monitoring.

Residual pharmaceuticals in water samples are determined with laboratory methods, such as fluid/gas chromatography coupled with mass spectrometry. Unfortunately, these methods are expensive (cost of devices + cost of analysis) and frequently, among others, relate to the very low concentration of analytes in water samples (at ng L^−1^ or pg L^−1^ levels) and the costly and time-consuming initial sample preparation stage [64,65,66,67,68]. Compared to that, the voltammetric techniques are characterized by their low cost, simplicity of the analytic process, and the possibility to accumulate the analyte onto the surface of the working electrode before the appropriate electrode process, which eliminates the need to apply additional concentrating techniques (e.g., the extraction to solid phase) [69].

**Table 1:** The concentrations and removal rates of painkillers in the environmental matrices.

Drug	Excretion and Metabolites	WWTP Removal Rate (%)	Wastewater Influent (ng/L)	Wastewater Effluent (ng/L)	Surface Water (ng/L)
diclofenac	5–10% unchanged, metabolites: glucuronide, sulfate conjugates [49]	9–60 [50] 57.9 [47]	up to 302 [50] 191,000 [47]	1300–3300 [51] Up to 5450 [50] 10,000 [52] 80,000 [47]	up to 490 [50] 1200 [48] 1410 [53]
ibuprofen	1% unchanged Metabolites: (+)-2-40-(2-Hydroxy-2-methylpropyl)-phenylpropionic acid (25%) and (+)-2-40-(2- carboxypropyl)-phenylpropionic acid (37%), conjugated ibuprofen (14%) [49]	78–100 [50] 94.8 [47]	5533 [50] 344,000 [47]	711 [50] 18,000 [47]	400 [50] 126 [53]
naproxen	<1 unchanged, metabolites: 6-o-Desmethyl naproxen (o1%), conjugates (66–92%) [49]	50–98 [50]	611,000 [50]	33,900 [50] 10,000 [52]	297 [53] 390 [48] 400 [50]
ketoprofen	Metabolites: Glucuronide conjugates [49]	15–100 [50]	5700 [50] 1000–10,000 [54]	1620 [50]	120 [48] 329 [50]
paracetamol	80% as conjugates, metabolites: Sulphate conjugate (30%), paracetamol cysteinate, mercapturate (5%) [49]	91–99 [50]	292,000 [50] 1000–10,000 [54]	1480 [50] 100,000 [52]	10,000 [48] 66 [50]
acetylsalicylic acid	Metabolites: Salicylic acid (10%), salicyluric acid (75%), salicylic phenolic (10%) and acyl (5%) glucuronides, gentisic acid (o1%) [49]	0 [50]	1000–10,000 [54]	1510 [50]	<50 [50]

In the 1990s, screen printing technology for the preparation of electrochemical sensors was introduced. The screen-printed electrodes (SPEs) became objects of numerous research efforts aimed at investigating their practical application. The low manufacturing costs, appropriate repeatability levels, and electrochemical properties, all make them an attractive analytical tool [70,71,72,73,74,75,76,77].

**Figure 1:**
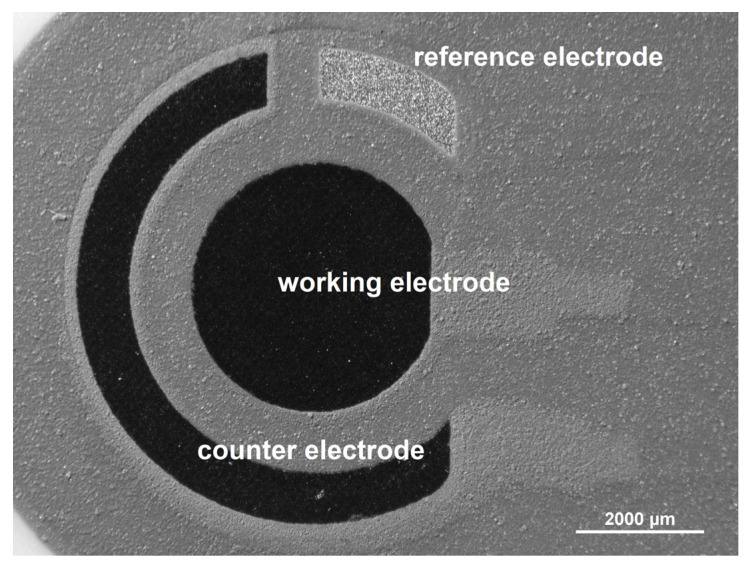
Optical microscopic image of screen-printed carbon electrode (SPCE, Metrohm DropSens, Oviedo, Spain).

The manufacture of screen-printed electrodes is the process of designing appropriate ink (print) composition and then pressing it through the appropriate template (screen) onto the carrier surface (most often ceramic or polymer). Both the ink composition and the area of the working electrode can be modified, e.g., with nanoparticles/metal films, polymer, or enzyme, depending on the application-specific requirements [70,71,72,73,74,75,76,77]. The entire electrode system (reference, counter, and working electrodes) is printed on the same substrate surface (Figure 1).

The key feature of the screen-printed electrodes, among other electrochemical sensors, is their miniaturization, enabling them to apply in portable/field devices. The application of the screen-printed electrodes in the carrying out measurements in situ enable minimalization or even elimination of errors, reduces the test time, and consequently, costs usually connected with sampling, transport, and storage of representative samples [78]. This justifies the thesis saying that there is a significant need to develop field devices for monitoring waters that will allow us to evaluate water quality at the sampling location in an easy and fast manner. Recent years saw the growing interest in the development of such devices. The use of portable devices is one of the development trends in environmental analytics [79].

A lot of papers describe the application of electrochemical sensors for the determination of residues of pharmaceuticals and screen-printed sensors [74,75,80]. Most electrochemical methods allow for the quantitative determination of these compounds in pharmaceutical preparations, biological samples, and beverages. However, the literature has available articles on the development of electrochemical sensors to determine pharmaceuticals, including painkillers in water samples. This article focuses on a summary of achievements in the field of screen-printed voltammetric sensors application in environmental water monitoring of painkillers.

## 2. Application of Screen-Printed Voltammetric Sensors for the Painkillers Determination in the Environmental Water Samples

**Figure 2:**
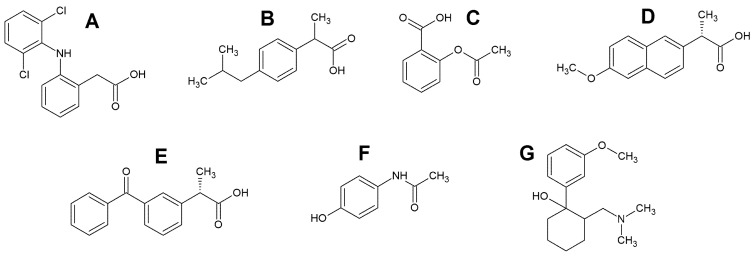
The structural formulas of diclofenac (**A**), ibuprofen (**B**), acetylsalicylic acid (**C**), naproxen (**D**), ketoprofen (**E**), paracetamol (**F**) and tramadol (**G**).

Non-steroidal anti-inflammatory drugs (NSAIDs) are an important class of drugs because they are widely used to treat muscle pain and inflammatory rheumatic diseases, and this is an increasing trend. This fact, combined with improper disposal and ineffectiveness of wastewater treatment, leads to the ubiquitous presence of these drugs in the environment [81]. Diclofenac (DF), ibuprofen (IB), acetylsalicylic acid (AS), naproxen (NP) and ketoprofen (KP) belong to the NSAIDs (Figure 2A–E). Diclofenac exhibits activities characteristic of this group of drugs, i.e., anti-inflammatory, antipyretic, analgesic, and inhibiting platelet aggregation [82,83]. It is used to relieve symptoms of many illnesses, including non-articular rheumatism, osteoarthritis, sports injuries, and rheumatoid arthritis. In the proposed daily dose (50–150 mg), DF is completely tolerated [84]. Although no problems are caused when an appropriate amount of DF is used, its excessive or continuous use may cause symptoms such as epigastric discomfort, gastric ulcer, hematuria; meantime, the accumulated mass of toxic substances can cause kidney and liver dysfunctions [85]. Furthermore, the ubiquity of DF in the environment impairs fish health and water quality due to its poor degradation [86]. Ibuprofen ( (2–4 isobutyphenyl) propionic acid is commonly prescribed to treat chronic and acute pain and many rheumatic and musculoskeletal disorders. IB is also used to reduce fever [87]. Its action is due to the inhibition of cyclo-oxygenases, which are involved in the synthesis of prostaglandins involved in producing pain, inflammation, and fever [88]. Acetylsalicylic acid (aspirin) is an anti-inflammatory, antipyretic, and analgesic drug. Thanks to its effectiveness, it has a place in treating antithrombotic coronary heart disease, prevention of colon cancer, fever, headaches, and Alzheimer’s disease [89,90]. Naproxen (2-(6-methoxy-2-naphthyl)propionic acid) is an antipyretic and anti-inflammatory compound applied in the treatment of nonrheumatic inflammation, migraine, and gout [91]. Association therapy with paracetamol and naproxen has also been reported to benefit patients with pain related to rheumatoid arthritis. The naproxen drug should be given with precaution to elderly patients and patients with hemophilia, gastrointestinal bleeding, and platelet coagulation dysfunction. [92]. Ketoprofen is an arylpropionic acid derivative with anti-inflammatory, antipyretic, and analgesic properties [93]. It relieves pain associated with rheumatic and nonrheumatic inflammatory disorders, vascular headaches, and dysmenorrhea [94]. It is well absorbed after oral and rectal administration, and it can be administered by injection and transdermally. KP is metabolized in the liver and mainly excreted in urine [93].

Paracetamol (N-acetyl-p-aminophenol, Figure 2F), also known as acetaminophen or Tylenol, is extensively used to relieve moderate pain and reduce fever globally [95]. It has no anti-inflammatory effect. PA is the main ingredient in many cold and flu medications [82]. The antipyretic effect of this drug is related to the inhibition of prostaglandin synthesis in the central nervous system [83]. An overdose of PA may result in the accumulation of toxic metabolites that can cause acute and sometimes fatal nephro- and hepatotoxicity [82]. As a widely used pharmaceutical, PA is present in the environment, its concentration found in environmental water samples ranges from 1 to 10 nM.

Tramadol, (1R,2R)-2-[(dimethylamino)methyl]-1-(3-methoxyphenyl)cyclohexanol, TR, Figure 2G, is an l-opioid recipient agonist that acts on analgesic centrally is applied initially for treating modest to severe pain [96]. TR is a commonly misused drug that can lead to addiction or even death, although it has a preferable safety profile than other opioid analgesic drugs such as morphine or hydrocodone [97]. The typical dosage requirement for oral consumption of TR ranges between 50 and 100 mg per 4 to 6 h. The maximal dose of the drug will be 400 mg every day. Overdosing of TR can cause nausea, respiratory depression, vomiting, coma, dizziness, and tachycardia.

In the literature are few articles describing the use of screen-printed sensors in the monitoring of painkillers residues (DF, PA, IB, and TR) in environmental waters samples [82,83,98,99,100,101,102,103,104]. These sensors have also found application in the determination of painkillers in pharmaceutical preparations and biological samples, e.g., urine and serum [84,85,86,87,96,97,105,106,107,108,109,110,111,112,113]. There are no studies on voltammetric procedures to determine acetylsalicylic acid and naproxen in water samples in the literature. However, there are single procedures for determining AS [89,90,114] and NP [92,115] on screen-printed electrodes in pharmaceuticals and/or human physiological fluids. There are no attempts to use screen-printed sensors to analyze ketoprofen in any type of sample.

### 2.1. SPEs Modified with Carbon Nanomaterials

Carbon nanomaterials are very attractive for the mass production of SPEs and represent a significant opportunity to increase the analytical sensitivity of these devices, enabling new sensing applications [116,117]. These materials are characterized by excellent electrical conductivity, low electrical resistance, large surface area, and good physical and chemical stability [118]. In addition, the possibility of functionalization of their surface leads to an increase in analytical efficiency, including sensitivity and selectivity [119]. Carbon nanomaterials used as SPEs modifiers include mainly carbon black (CB), graphene-related materials, carbon nanofibers (CNFs), various forms of carbon nanotubes—single-, double-, and multiwalled (SWCNTs, DWCNTs, and MWCNTs), carbon nanohorns (CNHs), as well as carbon nano-onions (CNOs) [117,120,121,122,123].

In the literature, few articles describe the use of screen-printed sensors modified with carbon materials to monitor painkillers residues in environmental water samples [83,98,99,101,102]. The comparison of these assay procedures is presented in Table 2. The lowest detection limit of paracetamol for the accumulation time of 90 s (LOD, 0.54 nM) was obtained using a screen-printed carbon/carbon nanofibers sensor (SPCE/CNFs) [98]. Sasal et al. [98] described the application of a commercially available SPCE/CNFs sensor and differential pulse adsorptive stripping voltammetry (DPAdSV) for the direct determination of the low (real) concentration of PA in environmental water samples. This was done by monitoring the oxidation current of PA after adsorption of molecules onto the SPCE/CNFs surface at the potential of −0.95 V. According to the literature data, a PA oxidation mechanism at the SPCE/CNFs is associated with the formation of N-acetyl-p-quinoneimine (NAPQI) [99]. The adsorption of PA onto the electrode surface was confirmed by electrochemical impedance spectroscopy (EIS), cyclic voltammetry (CV), and theoretical studies. Moreover, the authors found that the SPCE/CNFs sensor presented better performance than the screen-printed sensors with carbon or carbon/multiwalled carbon nanotubes working electrodes. It was related to the developed active surface of the SPCE/CNFs, which mediates PA adsorption. Under optimized conditions, the DPAdSV measurements were performed in 0.1 M H_2_SO_4_ containing 0.01 µM EDTA to minimize the influence effect of interfering metal ions. The developed analytical procedure using SPCE/CNFs was applied to the direct determination of PA (in the range of 5–200 nM) in water samples collected from two Polish rivers and sea with the recovery values between 96.2 to 104.6%.

**Figure 3:**
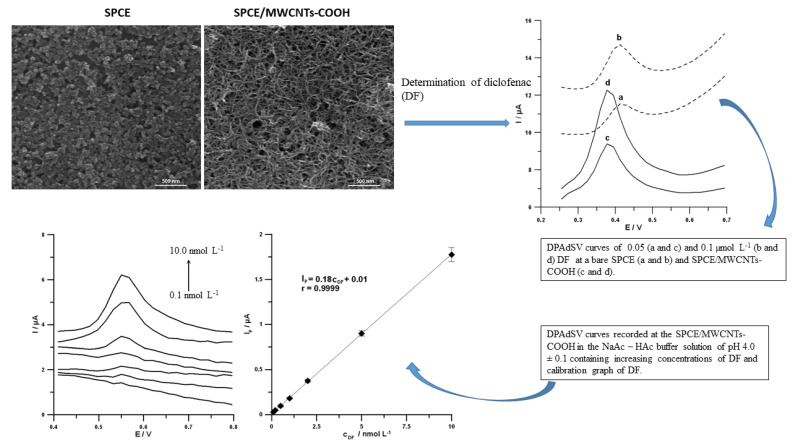
SEM images and DPAdSV curves recorded at the SPCE and SPCE/MWCNTs-COOH. DPAdSV curves recorded at the surface of the SPCE/MWCNTs-COOH in solution containing increasing concentrations of DF: 0.1, 0.2, 0.5, 1.0, 2.0, 5.0 and 10.0 nmol L^−1^, and calibration graph of DF [102].

In [102], Sasal et al. proposed a DPAdSV voltammetric procedure at the commercially available screen-printed carbon sensor modified with carboxyl functionalized multiwalled carbon nanotubes (SPCE/MWCNTs-COOH) for the trace analysis of diclofenac (DF) (Figure 3). The authors stated that DF is irreversibly oxidized, and the oxidation process of DF is not purely diffusion-or adsorption-controlled at the SPCE/MWCNTs-COOH. Moreover, the number of electrons involved in the DF oxidation process equals 2. These results are consistent with the literature data, which proposed that DF is oxidized to 5-hydrohydiclofenac by losses of 2e^−^ and 2H^+^ [124,125]. The SPCE/MWCNTs-COOH provided a higher sensitivity and wider linear range than the SPCE (0.019 vs. 0.040 µA/nM and 0.5–200.0 vs. 1.0–200 nM, respectively). It is connected with the fact that the application of MWCNTs-COOH improved the electron transfer process and the active surface area of the electrode. The proposed DPAdSV procedure is characterized by simplicity, sensitivity, and time-saving. For the first time, the electrochemical sensor was applied to determine the real concentration of DF (0.42 ± 0.080 nmol L^−1^) in the river water samples without the sample pre-treatment step.

**Figure 4:**
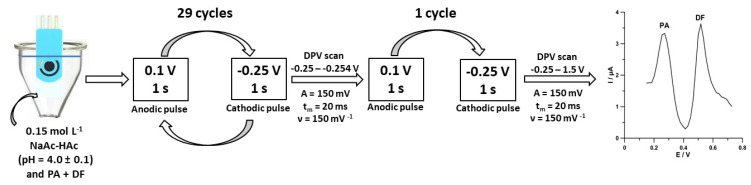
Scheme of voltammetric measurements of PA and DF at the SPCE/MWCNTs-COOH [83].

In voltammetric measurements, even a low concentration of surface-active substances can block the active surface of the electrode. Therefore, UV irradiation or microwave heating of the samples are suggested for the elimination of this type of interference. There are also other simple ways to minimize interferences from the organic matrix of samples, e.g., the application of potential pulses for accumulation. In that approach, the potential of cathode pulses was chosen in a way that made it represent the maximum adsorption of the determined element and the potential of anode pulses to desorb the interfering surfactants [126]. This method of eliminating interference from surfactants was described by Sasal et al. in [83]. The authors proposed using the DPAdSV technique, a commercially available SPCE/MWCNTs-COOH sensor, and pulsed potential accumulation for individual and simultaneous determination of paracetamol and diclofenac. The scheme of the individual steps of the optimized voltammetric procedure is presented in Figure 4. Moreover, the authors found that the application of carboxyl functionalized multiwalled carbon nanotubes and pulsed potential accumulation contributes to improving PA and DF peaks currents. The amplification of the PA and DF signals is related to a greater number of active centers at the SPCE/MWCNTs-COOH than at the SPCE. Based on the cyclic voltammetric examination, it was found that the PA and DF are irreversibly oxidized at the SPCE/MWCNTs-COOH, and these processes are not purely diffusion or adsorption controlled. The DPAdSV procedure with SPCE/MWCNTs-COOH shows the low LODs of 1.4 nM for PA and 0.03 nM for DF. The DPAdSV procedure at the SPCE/MWCNTs-COOH was successfully applied for the simultaneous analysis of PA and DF in spiked river water samples with recovery values between 96.5% and 104.8%. Moreover, the voltammetric procedure proposed by Sasal et al. allowed for the direct determination of PA (24.3 ± 0.5 nM) and DF (3.7 ± 0.7 nM) in the wastewater samples purified in a sewage treatment plant.

Serrano et al. [99] compared the analytical performances of the commercially available screen-printed carbon electrode (SPCE), multiwalled carbon nanotubes modified screen-printed carbon electrode (SPCNTE), screen-printed carbon electrode modified with carbon nanofibers (SPCNFE), and screen-printed graphene electrode (SPGPHE) for the individual and simultaneous determination of ibuprofen, paracetamol, and caffeine. The authors suggest that the SPCNFE was the most suitable carbon-based electrode for the voltammetric determination of the selected analytes in water at trace levels. Moreover, the applicability of SPCNFE for the analysis of environmental water samples was demonstrated by the simultaneous determination of PA, IB, and CF in spiked tap water samples. The good recoveries (103.1, 99.5, and 97.6% for PA, IB, and CF, respectively) and reproducibility (RSD of 8.93, 0.96, and 8.63% for PA, IB, and CF, respectively) were obtained. In hospital wastewater samples, only PA (7.74 µM) was determined. The IB and CF were not detected since the studied hospital wastewater sample did not contain IB, and the concentration of CF was below the LOD obtained for the SPCNFE.

In paper [101], Deroco et al. described the use of carbon black as a modifying nanomaterial of SPEs. The application of CB film onto the SPE surface contributed to the improvement of electrocatalytic activity for the [Fe(CN)_6_]^3−/4−^ redox probe, and paracetamol and levofloxacin (LVF) signals. The authors stated that PA and LVF oxidation processes were fully controlled by diffusion, and these processes involved an equal number of electrons and protons. The developed sensor was successfully used to analyze PA (6.0, 10.0, and 70.0 µM) and LVF (2.0, 9.0, and 60.0 µM) in spiked river water samples with recovery values between 98.3 and 106.0%.

**Table 2:** Summary of voltammetric procedures to determine painkillers residues at the screen-printed electrodes modified with carbon materials in environmental water samples.

Electrode	Analyte	Method	Linear Range [µM]	LOD [µM]	Application	Ref.
SPCE/CNFs	PA	DPAdSV	0.002–0.05 0.1–2.0	0.00054	river water, sea water	[98]
SPCE/MWCNTs-COOH	DF	DPAdSV	0.0001–0.01	0.000028	river water	[102]
SPCE/MWCNTs-COOH	PA DF	DPAdSV (PPA)	0.005–5.0 0.0001–0.02	0.0014 0.000030	wastewater, river water	[83]
SPCE	PA	DPV	13.20–377.0	7.17	tap water, hospital wastewater	[99]
SPCNTE			2.64–33.70	0.66		
SPCNFE			1.98–33.70	0.66		
SPGPHE			3.31–23.20	0.66		
SPCE	IB		18.40–489.60	5.33		
SPCNTE			9.21–155.10	2.91		
SPCNFE			19.40–114.40	5.82		
SPGPHE			30.50–86.30	9.21		
SPCE	CF		24.70–480.0	7.21		
SPCNTE			20.60–480.0	6.18		
SPCNFE			61.80–330.0	2.06		
SPGPHE			15.50–44.80	4.63		
CB/SPCE	PA LVF	SWV	0.80–30.0 0.90–70.0	2.60 0.42	river water	[101]

PA–paracetamol; DF–diclofenac; IB–ibuprofen; CF–caffeine; LVF–levofloxacin; DPAdSV–differential-pulse adsorptive stripping voltammetry; PPA–pulsed potential accumulation; DPV–differential-pulse voltammetry; SWV–square-wave voltammetry; SPCE–screen-printed carbon electrode; SPCE/MWCNTs-COOH–carboxyl functionalized multiwalled carbon nanotubes modified screen-printed carbon electrode; SPCNTE–screen-printed carbon electrode modified with carbon nanotubes; SPCE/CNFs (SPCNFE)–screen-printed carbon electrode modified with carbon nanofibers; SPGPHE–screen-printed graphene electrode; CB/SPCE–screen-printed carbon electrode modified with carbon black.

### 2.2. SPEs Electrochemically Pretreated

SPEs consist of working electrodes made of conductive inks based on platinum, gold, silver, or carbon, the latter being the most used material because it is universal and cheap. Conductive inks from screen-printed carbon electrodes (SPCEs) contain carbon with organic solvents, binding pastes (e.g., polyester resin, ethylcellulose, or epoxy-based polymeric binder), and some additives that provide functional properties. The presence of these additional non-conductive materials can lead to a slowdown in the kinetics of heterogeneous electrochemical reactions. Therefore, much attention has been paid to developing surface treatment methods to improve the electrochemical properties of the SPCEs. The main purpose of the SPCEs pre-treatment was to remove organic ink components or contaminants and increase surface roughness or functionality [127,128,129]. Several methods of pre-treatment of SPEs can be found in the literature, such as heat treatment [127], oxygen plasma treatment [128], chemical treatment [129], polishing [130,131], and electrochemical treatment [132,133,134]. Electrochemical treatments allow the in situ easy activation of SPCEs. They usually hold the electrode at a constant potential for a short time or potential cycling to extreme anodic and/or cathodic potentials [127,135]. Table 3 shows summary of voltammetric procedures for painkillers residues determination at the electrochemically pretreated screen-printed electrodes electrochemically. 

In paper [82], Kozak et al. proposed an electrochemically activated (25 voltammetric cycles from 1.0 to −0.7 V at a scan rate of 10 mV s^−1^ in a solution containing 0.1 M acetate buffer of pH = 4.0 ± 0.1 and 10 mM H_2_O_2_) screen-printed carbon electrode modified with sodium dodecyl sulfate (aSPCE/SDS) for the simultaneous determination of paracetamol, diclofenac, and tramadol. At the moment, there is the first paper describing the voltammetric procedure of tramadol determination with the use of screen-printed electrodes in environmental water samples [82]. The electrochemical activation contributes to the removal of the organic ink constituents or contaminants introduced into the printing stage, which consequently changes the morphology of the electrode surface and reduces the charge transfer resistance. Furthermore, the modification of the electrode surface with SDS contributes to the TR signal amplification and minimizes the influence of surfactants (Triton X-100 and CTAB). The author proved the adsorption of PA and DF onto the SPCE/SDS surface by analyzing differential capacity curves and stated that TR existing in the cationic form reaches the electrode surface by diffusion and is electrostatically attractive for the surface covered by SDS anions. The aSPCE/SDS showed a good linear response in the concentration ranges of 0.05–2.0 µM for PA, 0.001–0.2 µM for DF, and 0.01–0.2 and 0.2–2.0 µM for TR. The limits of detection obtained during the simultaneous determination of PA, DF, and TR are 0.015 µM, 0.00021 µM, and 0.0017 µM, respectively. The DPAdSV procedure with the aSPCE/SDS was successfully applied for the determination of PA, DF, and TR in river water and serum samples (the recovery values between 97.0 and 102.0%) as well as pharmaceuticals (the relative errors between determined and label values are in the ranges of 0 and 2.1%).

In paper [100], Raymounds-Pereira et al. proposed electrochemically pretreated (2 voltammetric cycles from −2.5 to 2.5 V at a scan rate of 100 mV s^−1^ in 0.5 M sulfuric acid solution) screen-printed carbon electrode (SPCE) for the determination of hydroquinone (HQ), paracetamol and estradiol (E2) in tap water. The authors concluded that pre-treatment did not affect the morphology of the electrode surface and did not introduce functional groups to the surface but removed non-conducting residues from the printing ink. This consequently contributed to the improvement of the sensor’s conductivity (the charge-transfer resistance decreased from 30 to 5 kΩ due to the pre-treatment) and sensitivity of the voltammetric procedure. The ability to determine PA, HQ, and E2 was verified by taking voltammetric measurements with tap water samples spiked with analytes in the concentration range of 1–7 µM). The results were compared to those obtained by high-performance liquid chromatography (HPLC). Using the Student’s test the author found that the voltammetric procedures yielded the same results as the standard method HPLC.

Amin et al. [104] show the application of electrochemically pretreated screen-printed graphite electrode (SPGE) to the electrochemical oxidation and detection of ibuprofen (IB). An SPGE surface was pretreated (conditioned) by applying a fixed potential of 1.6 V for 3 min vs. Ag pseudo reference electrode. Using pretreated SPGE, detection limits were improved 12.5 times to instrumental detection limits, thereby, LOD of 6.30 µM was achieved.

### 2.3. SPEs Modified with Polymers

Polymers are widely used modifiers for electrodes and SPEs. The most frequently used polymers for this purpose are conductive polymers that combine conventional properties of polymers with the electronic properties of metals and/or semiconductors. Mainly polyacetylene, polyaniline, polypyrrole, or polythiophene are used here [136,137]. In addition, it was found that the use of a conductive polymer to modify the electrode surface increases the electrical conductivity, high chemical stability, good magnetic properties, high electron affinity, optical properties, and low ionization potential [138]. Another group of polymers that can be used to modify the surface of the electrodes are ion-exchange polymers, one example of which is the well-known Nafion–perfluorinated sulfonated cation-exchanger [138]. Today, molecularly imprinted polymers (MIPs) in combination with electrochemical sensors are of great interest. MIP modified electrodes have proven the usefulness of both small and large biomolecules, such as proteins or DNA [139,140]. MIPs are typically prepared by forming a three-dimensional polymer network around a molecular template via a cross-linking step. Removal of this matrix creates binding cavities that retain the shape, size, and orientation of the target molecule, leading to a high selectivity in the recognition process [141,142]. MIPs offer some clear advantages, including very good stability with a high surface area over a wide range of experimental conditions and solvents, becoming powerful alternatives to biorecognition elements such as antibodies. Because of their high selectivity, simple synthesis methods, high stability, low cost, and good engineering capability, MIPs receive great attention as recognition elements in various fields, especially in electrochemical sensing [143,144]. The polymer layer on the surface of the electrodes can be applied in several ways. Most often it is done by dropping a polymer solution [145] or electropolymerization [137,146,147]. Other methods described in the literature are dispensing, inkjet printing, screen-printing, electrodeposition, electrospray, or pen-writing [138].

Seguro et al. [103] proposed a disposable voltammetric molecularly imprinted polymer screen-printed carbon sensor (MIP/SPCE) for the selective determination of diclofenac (Table 3). MIP preparation was achieved by cyclic voltammetry, using dopamine as a monomer in the presence of DF. The MIP/SPCE showed adequate selectivity (in comparison with other drug molecules), intra-day repeatability of 7.5%, inter-day repeatability of 11.5%, a linear range between 0.1 and 10 µM, and a limit of detection and quantification of 70 and 200 nM, respectively. Its applicability was demonstrated by the determination of DF in spiked water samples (river and tap water).

## 3. Conclusions

Monitoring the water environment for the presence and content of residues of pharmaceuticals, including painkillers a significant issue for contemporary analytical chemistry. This review demonstrates the applications of screen-printed sensors for the sensitive determination of environmental water pollutants (painkillers). Several examples described in this review paper show that the developed simple, sensitive, and selective voltammetric procedures with screen-printed sensors can be good tools for this purpose. The screen-printed electrodes modified with carbon nanomaterials, polymer film, or electrochemically activated showed advantageous electroconductivity, catalytic activity, and surface area. Moreover, the screen-printed sensors are potentially applicable not only in laboratory measurements but also in-field analysis. Due to their electrochemical properties, simplicity, disposability, short response time, and miniaturization, screen-printed sensors can find application in environmental water monitoring.

The screen-printed sensors area is expected to grow with new application domains. Future work will focus on improving the analytical parameters of screen-printed sensors to adjust them to the relative concentrations of the analytes in the environmental water samples, and on developing procedures and sensors for new substances AS, NP, KP) with analgesic properties. More attention should be given to expected interfering species and proven or possible strategies for mitigating their effects and improving selectivity. Moreover, sensor miniaturization, shortening the analysis time, reducing the volume of analyzed samples, and using reagents should be the aim of subsequent studies.

## Figures and Tables

**Table 3:** Summary of voltammetric procedures for painkillers residues determination at the electrochemically pretreated screen-printed electrodes or modified with polymer film in environmental water samples.

Electrode	Analyte	Method	Linear Range [µM]	LOD [µM]	Application	Ref.
aSPCE/SDS	DF PA TR	DPAdSV	0.001–0.2 0.05–20.0 0.01–0.2 0.2–2.0	0.00021 0.015	river water	[82]
electrochemically pretreated SPCE	PA HQ E2	DPV	0.5–10.0 0.5–10.0 0.5–10.0	0.22 0.19 0.89	tap water	[100]
electrochemically pretreated SPGE	IB	SWV	0.80–30.0	6.30	river water, wastewater	[104]
MIP/SPCE	DF	DPV	0.1–10	0.07	river water, tap water	[103]

PA–paracetamol; DF–diclofenac; HQ–hydroquinone; E2–estradiol; IB–ibuprofen; DPAdSV–differential-pulse adsorptive stripping voltammetry; DPV–differential-pulse voltammetry; SWV–square-wave voltammetry; aSPCE/SDS–activated screen-printed carbon electrode modified with sodium dodecyl sulfate; electrochemically pretreated SPCE–electrochemically pretreated screen-printed carbon electrode; electrochemically pretreated SPGE–electrochemically pretreated screen-printed graphite electrode; MIP/SPCE–screen-printed carbon electrode modified with molecularly imprinted polymer.

## Data Availability

The data presented in this study are available on request from the corresponding author.
